# Thematic trends and knowledge-map of tumor-infiltrating lymphocytes in breast cancer: a scientometric analysis

**DOI:** 10.3389/fonc.2024.1438091

**Published:** 2024-11-01

**Authors:** Jinan Shi, Lei Pan, Feixia Ma, Ganlu Zhang, Yin Duan

**Affiliations:** ^1^ Department of Medical Oncology, Zhejiang Hospital, Hangzhou, Zhejiang, China; ^2^ Department of Breast Surgery, The First Affiliated Hospital of Zhejiang Chinese Medical University (Zhejiang Provincial Hospital of Chinese Medicine), Hangzhou, Zhejiang, China

**Keywords:** breast cancer, tumor-infiltrating lymphocytes, scientometric analysis, Bibliometrix, co-word analysis, hotspot

## Abstract

**Background:**

Tumor-infiltrating lymphocytes (TILs), essential for the anti-tumor response, are now recognized as promising and cost-effective biomarkers with both prognostic and predictive value. They are crucial in the precision treatment of breast cancer, particularly for predicting clinical outcomes and identifying candidates for immunotherapy. This study aims to encapsulate the current knowledge of TILs in breast cancer research while evaluating research trends both qualitatively and quantitatively.

**Methods:**

Publications on TILs in breast cancer studies from January 1, 2004, to December 31, 2023, were extracted from the Web of Science Core Collection. Co-occurrence and collaboration analyses among countries/regions, institutions, authors, and keywords were performed with Bibliometrix R packages and VOSviewer software. CiteSpace was used for reference and keyword burst detection, while high-frequency keyword layouts were generated using BICOMB. gCLUTO was employed for biclustering analysis of the binary co-keyword matrix.

**Results:**

A total of 2,066 articles on TILs in breast cancer were identified. Between 2004 and 2023, the USA and Milan University led productivity in terms of country/region and institution, respectively. The journals “CANCERS,” “Breast Cancer Research and Treatment,” and “Frontiers in Oncology” published the most articles on this topic. Loi S was the leading author, with the highest number of publications and co-citations. Co-keyword analysis revealed six research hotspots related to TILs in breast cancer. The pathological assessment of TILs using artificial intelligence (AI) remains in its early stages but is a key focus. Burst detection of keywords indicated significant activity in “immune cell infiltration”, “immune checkpoint inhibitors”, and “hormone receptor” over the past three years.

**Conclusion:**

This study reviews recent advancements and trends in TILs research in breast cancer using scientometric analysis. The findings offer valuable insights for funding decisions and developing innovative strategies in TILs research, highlighting current research frontiers and trends.

## Introduction

Breast cancer (BC) is the foremost cause of cancer deaths among women and ranks as the second most frequent cancer following lung cancer globally (1, 2). Clinically, BC is divided into four subtypes determined by the expression levels of estrogen receptor (ER), progesterone receptor (PR), and human epidermal growth factor receptor 2 (HER2).Standard treatments entail chemotherapy, hormone therapy, and targeted therapy, tailored to the subtype. Immunotherapy is now a primary focus for researchers worldwide, owing to its variety of strategies, particularly immune checkpoint inhibitors (ICIs) (3).

The survival, growth, invasion, and metastasis of BC cells are profoundly impacted by the tumor microenvironment (TME) (4). Tumor-infiltrating lymphocytes (TILs), as the most important component of TME, are vital for mediating adaptive immune responses against cancer cells (5). In BC, the predominant TIL subtypes include CD8+, CD4+, FOXP3+ T cells, and CD19+ B cells, whereas CD56+ NK cells, macrophages, and dendritic cells (DCs) are less frequently observed (6). The majority of TILs are located in the stromal region next to the tumor, termed stromal TILs (sTILs). A smaller proportion of TILs is found within the tumor itself, known as intratumoral TILs (iTILs) (7). Approximately 10% of luminal A/B BCs exhibit detectable TILs, while the prevalence is 15% in HER2-positive breast cancer and 20% in triple-negative breast cancer (TNBC) (6).

The structural composition of TILs in BC may have both prognostic and predictive significance. Enhanced lymphocyte infiltration is typically associated with improved outcomes across all BC subtypes, particularly in TNBC and HER2-positive BC (8). For example, a higher presence of CD8+ cells in TILs before therapy, as well as in the lymphoid infiltrate in the tumor bed after neoadjuvant treatment, is correlated with an increased pathological complete response (pCR) rate (9). Furthermore, immune checkpoint molecules like PD-L1 expressed by tumor cells can inhibit TIL activity and aid in immune evasion (10). Therefore, the density of TILs may serve as a potential predictive marker in immunotherapy (11). The levels and composition of TILs can also be impacted by prior treatments, such as neoadjuvant therapy, which may modify the number of TILs in residual disease, typically leading to a decreased amount of FOXP3+ cells (12, 13). In BC, lymphocytic infiltrates can be found in both primary tumors and metastatic sites. However, metastatic locations generally show fewer TILs compared to primary tumors, and the prognostic significance of TILs infiltration in metastatic lesions is still uncertain (14). Although numerous studies on TILs in breast cancer have provided some understanding, a thorough analysis of the overall trends in this field is yet to be performed.

Scientometrics utilizes both mathematical and statistical techniques to quantitatively evaluate the landscape of scientific research, covering various topics and trends by examining aspects such as country, institution, and authorship, among others (15). In this study, bibliometric methods are applied to perform a comprehensive quantitative and qualitative evaluation of TILs research in BC over the last two decades. This bibliometric study aims to analyze the research landscape of TILs in BC. Our analysis focuses on: publication trends and patterns in TILs research related to BC, identification of key authors, institutions, and countries contributing to this field, analysis of the most influential papers and their impact, exploration of research hotspots and emerging trends in TILs and BC studies.

## Materials and methods

### Data source and search strategy

This research relied on the Web of Science Core Collection (WoSCC) as its main data source, recognized throughout academia as a leading digital repository for scholarly literature and commonly used in bibliometric studies. The primary search terms were “breast cancer” and “tumor-infiltrating lymphocytes,” employing a strategy that included multiple synonyms and related terms to ensure comprehensive coverage: (TS=(“breast cancer*” OR “breast neoplasm*” OR “breast tumor*” OR “breast carcinoma*” OR “breast tumour*” OR “mammary cancer*” OR “mammary carcinoma*” OR “mammary neoplasm*” OR “mammary tumor*” OR “mammary tumour*”)) AND (TS=(“tumor-infiltrating lymphocytes” OR “tumor-infiltrating lymphocyte” OR “intratumoral lymphocyte*” OR “tumor-associated lymphocyte*” OR “tumor-invasive lymphocyte*” OR “lymphocyte* infiltrating tumor” OR “lymphocyte* within the tumor microenvironment” OR “tumor-infiltrating immune cell*”)).

A focused one-day search was conducted on March 7, 2024, to minimize biases from database updates. The search spanned all publication dates from January 1, 2004 to December 31, 2023. The study included only articles and reviews. Our analysis encompassed various study designs, including but not limited to randomized controlled trials, cohort studies, case-control studies, and basic scientific research, to provide a comprehensive overview of the field. The publications were limited to those written in English to ensure uniformity in language analysis.

### Data collection

**Figure 1:**
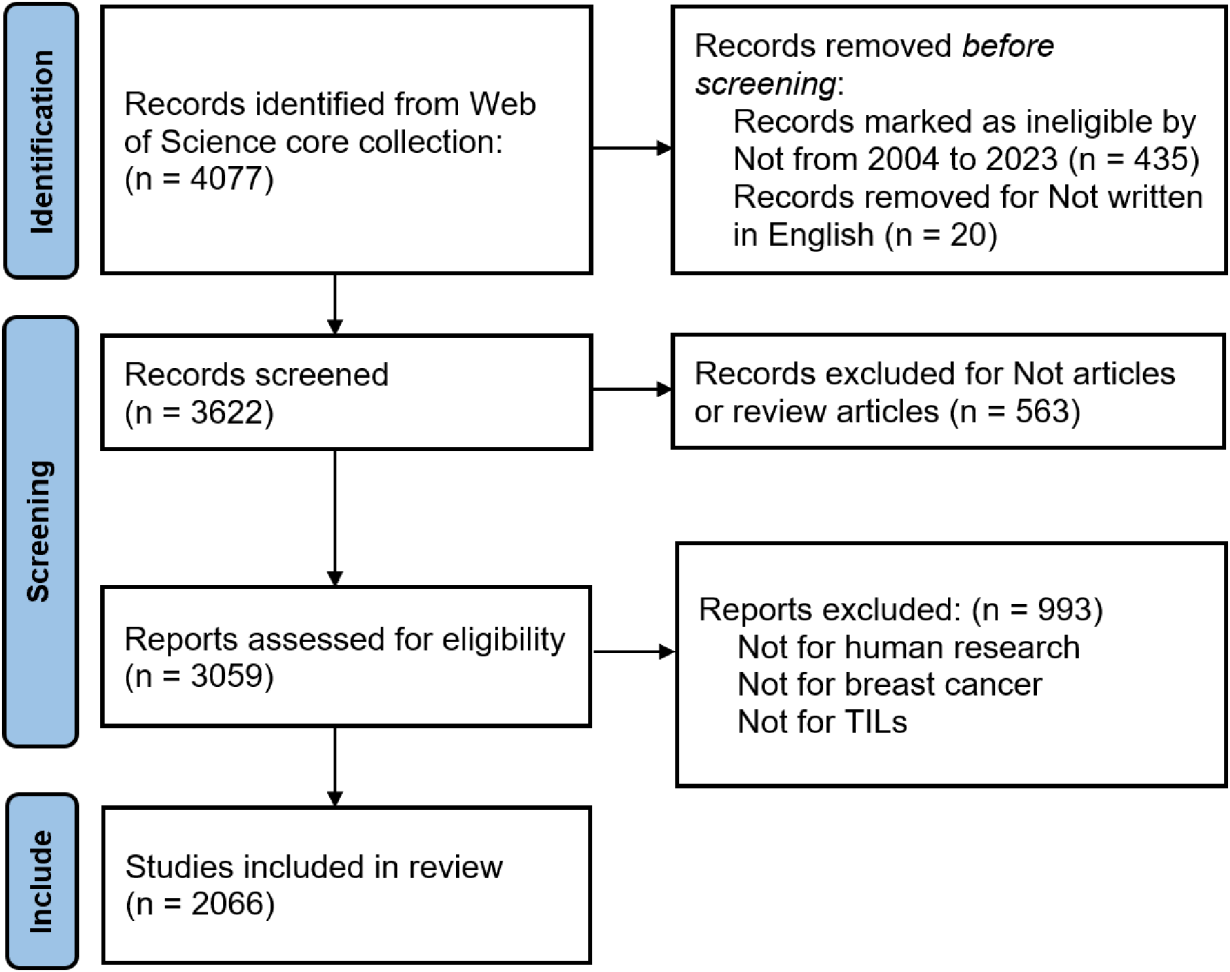
Flow Diagram of the Publication Selection Process.

Titles and abstracts were screened for relevance based on predefined criteria, specifically targeting publications related to TILs in BC. The retrieval process was independently conducted by two researchers, who resolved disagreements through discussion to achieve consensus. In cases of discrepancies during the evaluation, a third reviewer was brought in to make the final decision regarding data inclusion. The review included an analysis of each article’s title, publication date, author details and affiliations, journal, references, citation count, and widely used keywords, with the abstracts collected in “Plain Text File” format. The Preferred Reporting Items for Systematic Reviews and Meta-Analyses (PRISMA) guideline was used to conduct the systematic review, and bibliometric study presented here, and our procedure followed it unless stated otherwise. **Figure 1** succinctly illustrates the detailed data collection and inclusion procedures.

### Bibliometric analysis

Bibliometric analysis utilizes mathematical and statistical approaches to evaluate research findings, reveal insights, and identify developmental trends in various publications. By employing bibliometric mapping software for visual representations, researchers can thoroughly assess the scientific landscape, predicting upcoming trends and key areas within a specific field.

VOSviewer facilitates the visualization of research profiles, references, and geographical data, depicting productivity and impact across disciplines (16). It supports multiple co-occurrence analyses such as network, overlay, and density visualizations applied to various entities like countries, organizations, and authors. Using VOSviewer 1.6.20, we analyzed collaboration networks across different countries and institutions, producing a detailed graph where node sizes represent publication volumes or citation counts. Different colored nodes indicate specific clusters or periods, with lines showing connections such as collaborations or citations. Biblioshiny, a flexible R-based tool, integrates smoothly with diverse graphical and statistical functionalities. We imported data, including authors and cited references, as raw files into the R-4.2.3-based bibliometrics package, Bibliometricx. Biblioshiny helped extract crucial publication metrics, offering insights into total publications, annual scientific contributions, institutional impacts, author details, and word cloud. Developed in Java by Chaomei Chen, CiteSpace 6.3.R1 is a robust bibliometric tool known for revealing trends and key aspects within specific research areas (17). Its burst analysis function detects significant shifts in topics, offering critical insights. In our research, we conducted a burst analysis on cited references and keywords to spotlight seminal works. We also employed timeline visualization of keyword clusters to examine the distribution and evolution of topics. Finally, we used BICOMB, a text mining tool for biomedicine, to extract keywords from bibliographic databases to build co-occurrence keywords’ matrices. gCLUTO, a versatile graphical tool, clusters multi-dimensional datasets and analyzes their attributes. We extracted high-frequency keywords using BICOMB and analyzed them with gCLUTO’s co-word matrix to identify research hotspots. Furthermore, we generated heat maps and mountain maps to visually represent our findings.

## Results

### Overview of the key information

Utilizing data from Biblioshiny and integrating the “analyze results” tool from Web of Science, we conducted a preliminary evaluation to determine if the outcomes met comprehensive standards. From 2004 to 2023, our search strategy yielded 2,066 publications on TILs in BC, comprising 1530 articles and 536 review papers, that were included in the final analysis. These articles, published on average 4.4 years ago, received an average of 41.13 citations each. Collectively, these publications were cited 84,964 times, including 16,919 self-citations. Notably, international co-authorships constituted 27.3% of the totals, underlining a robust collaborative network across countries and regions within this research domain. We organized the data sources and descriptive statistics, detailed in **Supplementary Table 1.**

### Publication and citation trends over time

**Figure 2:**
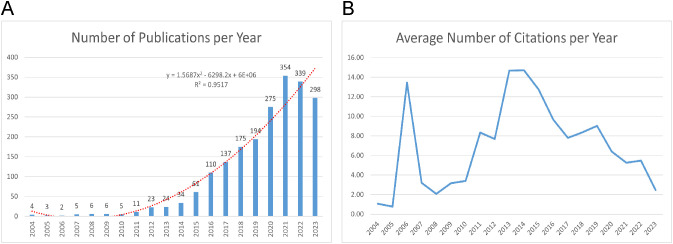
Publication and Citation Trends. **(A)** Annual publications output with polynomial fitting curve between 2004-2023. **(B)** The number of average citations per year (2004-2023).

**Figure 2A** displays a range in the number of published papers, with a minimum of 2 in 2006 and a maximum of 354 in 2021, reflecting an annual growth rate of 25.47%. The publication trends can be divided into two distinct phases. Between 2004 and 2013, the number of published articles consistently remained low, indicating a lack of significant trends in publication volume. Since 2014, however, a consistent rise in the volume of published articles is observed. Meanwhile, we utilized a polynomial fitting curve to clarify the observed trend, yielding a coefficient of determination (R²=0.9517) which underscores its statistical significance. **Figure 2B** shows the annual average citations per year for each artile, which follow a parabolic trend peaking in 2014 with an average of 14.71 citations. As a result, this field has attracted increasing attention, offering numerous opportunities for further research.

### Analysis of countries/regions and institutions

Research on TILs in BC has seen involvement from 86 countries. **Table 1** highlights the ten most active countries in this domain. The USA is the top contributor with 625 publications, followed by China with 428 and Italy with 228 publications. The USA also leads in total citations, amassing 37,971, which greatly exceed those of any other country. Conversely, China, despite its high publication count, has significantly fewer citations, with a total of 10,972.

**Table 1:** Top 10 countries with the most publications in the research field of TILs in BC.

Rank	Countries/Regions	Publications (%)	Total Citations	MCP/SCP
1	USA	625 (30.25%)	37971	0.412
2	PR China	428 (20.72%)	10972	0.097
3	Italy	228 (11.04%)	15781	0.472
4	Japan	152 (7.36%)	5186	0.110
5	Germany	139 (6.73%)	12605	0.775
6	France	137 (6.63%)	13702	0.689
7	Belgium	134 (6.49%)	14461	1.842
8	South Korea	130 (6.29%)	4208	0.063
9	Australia	116 (5.61%)	14458	1.308
10	Spain	116 (5.61%)	5716	0.590

MCP, Multiple Country Publications; SCP, Single Country Publications

**Figure 3:**
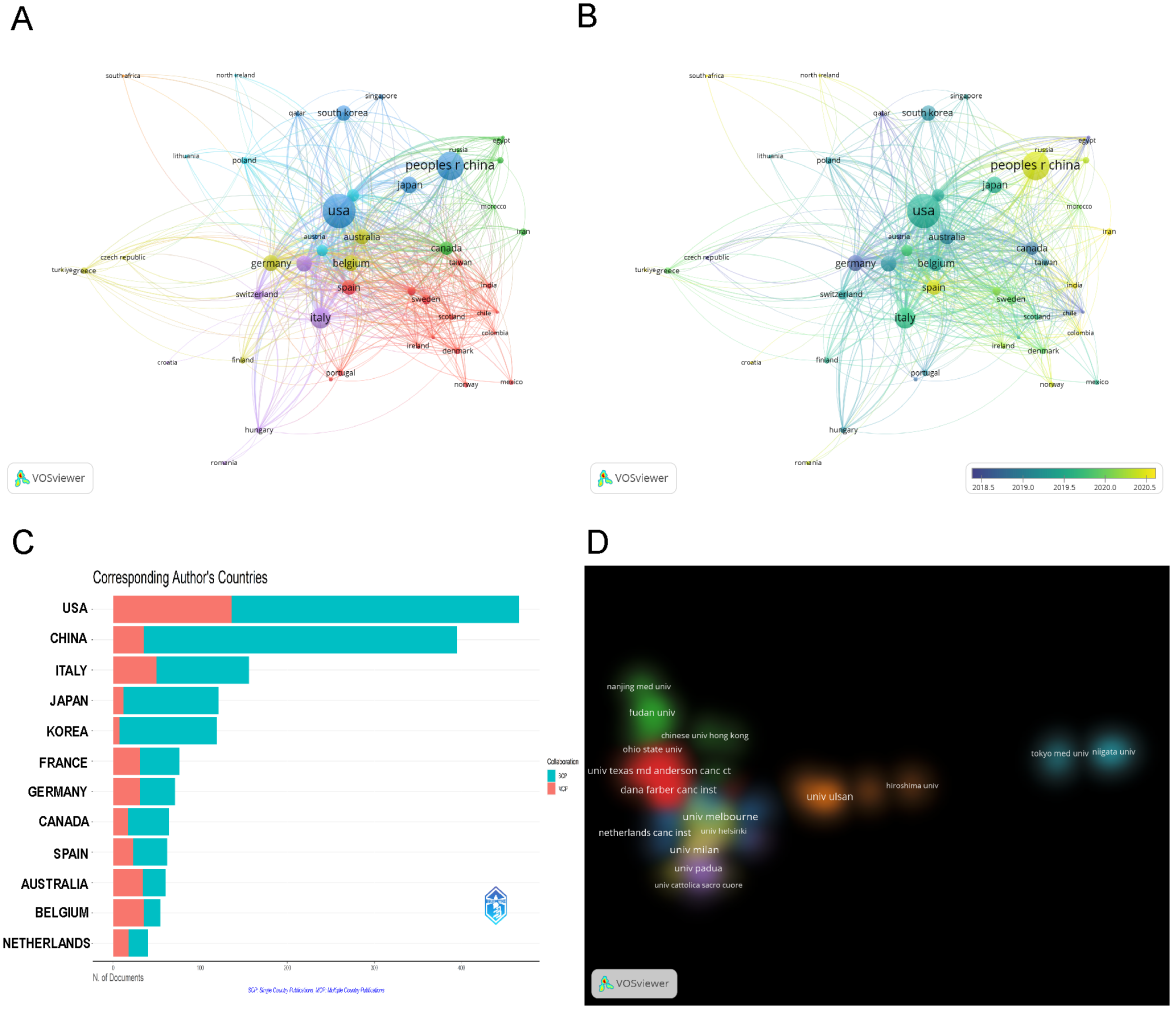
Visualization of Countries/Regions (Institutions) Analysis. **(A)** Visualization of the cooperation network map of countries/regions. **(B)** Visualization of the overlay map of countries/regions. **(C)** Cooperation of corresponding author’s countries (SCP, Single Country Publications; MCP, Multiple Country Publications). **(D)** Cluster density visualization map of institutions.

Fifty countries, each with over five publications, were analyzed for co-authorship, as shown in **Figure 3A**. The USA dominated in international collaborations, with a link strength of 655 spanning 46 countries. It had the strongest collaborations with Italy (link strength: 58), Belgium, and China (link strengths: 52 and 51, respectively). The overlay visualization with VOSviewer (**Figure 3B**) shows that Germany was an early leader, with an average publication year (APY) of 2018.63. It was followed by Belgium and the USA, with APYs of 2019.34 and 2019.43, respectively. Conversely, China and Spain joined the field later, with APYs of 2020.35 and 2020.39. In exploring the geographic distribution of corresponding authors, we differentiate between Single Country Publications (SCP) and Multiple Country Publications (MCP), which denote research conducted within one country and collaboratively across nations, respectively. The MCP ratio (MCP/SCP) detailed in **Figure 3C**, **Table 1** quantifies the extent of international collaboration. Belgium exhibited the most significant level of collaboration, with an MCP ratio of 1.842, ahead of Australia (1.308) and Germany (0.775).

Out of 3,117 research institutions, the leading five with the most publications include Milan University (n=60), Melbourne University (n=55), Peter Maccallum Cancer Center (n=51), Dana Farber Cancer Institute (n=47), and MD Anderson Cancer Center (n=47), detailed in **Table 2**. Additionally, co-authorship analysis encompassed 131 institutions, each with over ten publications. Using VOSviewer, inter-institutional collaborations were analyzed and depicted as density clusters within the co-authorship network. The study demonstrated that the iinsittution network consisted of seven distinct clusters, each marked by a unique color. As illustrated in **Figure 3D**, cluster 1 (red) is represented by Dana Farber Cancer Institute and MD Anderson Cancer Center, cluster 2 (green) Fudan University, cluster 3 (blue) Netherlands Cancer Institute, cluster 4 (yellow) Milan University and Melbourne University, cluster 5 (purple) Padua University, cluster 6 (cyan) Tokyo Medical University, cluster 7 (orange) Ulsan University.

**Table 2:** Top 10 institutions with the most publications in the research field of TILs in BC.

Rank	Institutions	Publications (%)	Total Citations
1	Univ Milan	60 (2.90%)	4227
2	Univ Melbourne	55 (2.67%)	5083
3	Peter Maccallum Canc Ctr	51 (2.47%)	7036
4	Dana Faber Canc Inst	47 (2.27%)	4108
5	Univ Texas MD Anderson Canc Ctr	47 (2.27%)	3894
6	Mem Sloan Kettering Canc Ctr	41 (1.98%)	3449
7	Univ Libre Bruxelles	40 (1.94%)	3411
8	Univ Ulsan	39 (1.89%)	1291
9	Harvard Med Sch	36 (1.74%)	985
10	Fudan Univ	36 (1.74%)	983

### Analysis of authors/co-cited authors

A total of 12,069 authors have contributed to research TILs in BC. **Table 3** displays the top ten most prolific and frequently co-cited authors. Loi S, with 43 publications, topped the list of ten authors by having the highest citation count of 5,416 and an H-index of 42. He was followed by Salgado R with 35 publications and Lee HJ with 34 publications. Among the co-cited authors, Loi S led with 1,934 co-citations, followed by Denkert C with 1,661, and Schmid P with 1,115.

**Table 3:** Top 10 authors (co-cited authors) with the most publications(co-citations) in the research field of TILs in BC.

Rank	Author	Publications	Citations	H-index	Co-cited Author	Co-citations
1	Loi S	43	5416	42	Loi S	1934
2	Salgado R	35	4201	34	Denkert C	1661
3	Lee HJ	34	893	20	Schmid P	1115
4	Gong G	33	887	19	Salgado R	1108
5	Sotiriou C	32	4375	29	Adams S	930
6	Curigliano G	28	1226	21	Dieci MV	723
7	Denkert C	24	2797	30	Emens LA	628
8	Pusztai L	23	1944	21	Loibl S	462
9	Park IA	20	690	16	Mittendorf EA	459
10	Dieci MV	19	1030	22	Nanda R	454

**Figure 4:**
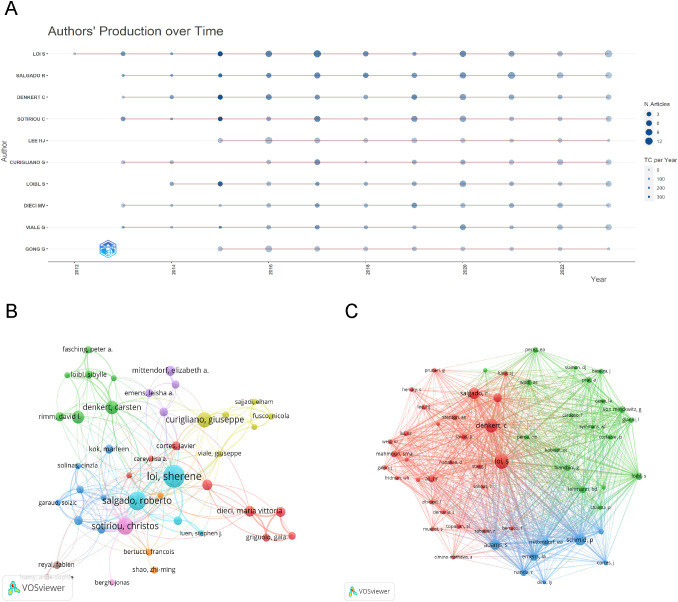
Visualization of Authors and Co-cited Authors Analysis. **(A)**Top 10 authors’ publications over time. **(B)** Visualization of the cooperation network map of authors. **(C)** Visualization of the network map of co-cited authors.

We employed Bibliometrix for the analysis of publication trends, concentrating on the ten most prolific authors. **Figure 4A** illustrates the node size reflecting the document count, with varying color shades denoting total citations (TC). Evidently, the majority of these authors demonstrated a sustained publication output over the previous decade.

Utilizing VOSviewer, we analyzed and depicted the author network, focusing on authors with a minimum of ten publications and a co-citation network comprising authors with over 150 co-citations. **Figure 4B** shows the collaboration networks among highly productive authors. Each color block’s size corresponds to the publication count, and the line thickness between authors reflects the volume of their collaborative works. Notably, the strongest collaboration occurs between Loi S and Salgado R, with a link strength of 20. **Figure 4C** depicts the co-citation network, being grouped into three clusters, color-coded as red (Cluster 1), green (Cluster 2), and blue (Cluster 3), with each cluster’s circle size reflecting the total link strength among its authors. In Cluster 1, Loi S shows a link strength of 26,473 and Denkert C 21,317; in Cluster 2, Loibl S registers at 9,358; and in Cluster 3, Schmid P stands at 18,456. This evidence clearly demonstrates active collaboration among authors and co-cited authors.

### Analysis of journals/co-cited journals and articles

Four hundred and eight journals have published articles on TILs in BC, with 47 of these journals publishing more than ten articles each. The journal CANCERS led with 131 publications, followed by BREAST CANCER RESEARCH AND TREATMENT with 84, FRONTIERS IN ONCOLOGY with 77, and CLINICAL CANCER RESEARCH with 66. **Table 4** lists the top 10 journals, with CLINICAL CANCER RESEARCH recording the highest impact factor at 10.1. Additionally, each of the top 10 co-cited journals was cited over 1800 times. The JOURNAL OF CLINICAL ONCOLOGY led with 9,430 co-citations, followed by the ANNALS OF ONCOLOGY with 6,121, and CLINICAL CANCER RESEARCH with 5,477. Among these, the NEW ENGLAND JOURNAL OF MEDICINE boasted the highest impact factor at 96.2, followed by NATURE at 50.5, JOURNAL OF CLINICAL ONCOLOGY at 42.1, and LANCET ONCOLOGY at 41.6.

**Table 4:** Top 10 Journalss (co-cited journals) with the most publications(co-citations) in the research field of TILs in BC.

Rank	Journal	Publications (%)	IF/JIF Quartile (2023)	Co-cited Journal	Co-citations
1	CANCERS	131 (6.60%)	5.2/Q2	JOURNAL OF CLINICAL ONCOLOGY	9360
2	BREAST CANCER RESEARCH AND TREATMENT	83 (4.18%)	3.8/Q2	ANNALS OF ONCOLOGY	6083
3	FRONTIERS IN ONCOLOGY	73 (3.68%)	4.7/Q2	CLINICAL CANCER RESEARCH	5371
4	CLINICAL CANCER RESEARCH	66 (3.32%)	11.5/Q1	CANCER RESEARCH	4434
5	BREAST CANCER RESEARCH	51 (2.57%)	7.4/Q1	NEW ENGLAND JOURNAL OF MEDICINE	3744
6	FRONTIERS IN IMMUNOLOGY	50 (2.52%)	7.3/Q1	BREAST CANCER RESEARCH AND TREATMENT	3701
7	BMC CANCER	41 (2.06%)	3.8/Q2	NATURE	2743
8	NPJ BREAST CANCER	40 (2.14%)	5.9/Q1	LANCET ONCOLOGY	2698
9	SCIENTIFIC REPORTS	40 (2.14%)	4.6/Q2	BREAST CANCER RESEARCH	2393
10	JOURNAL FOR IMMUNOTHERAPY OF CANCER	29 (1.46%)	10.9/Q1	PLOS ONE	1751

IF, Impact Factor; JIF Quartile, Journal Impact Factor.

**Figure 5:**
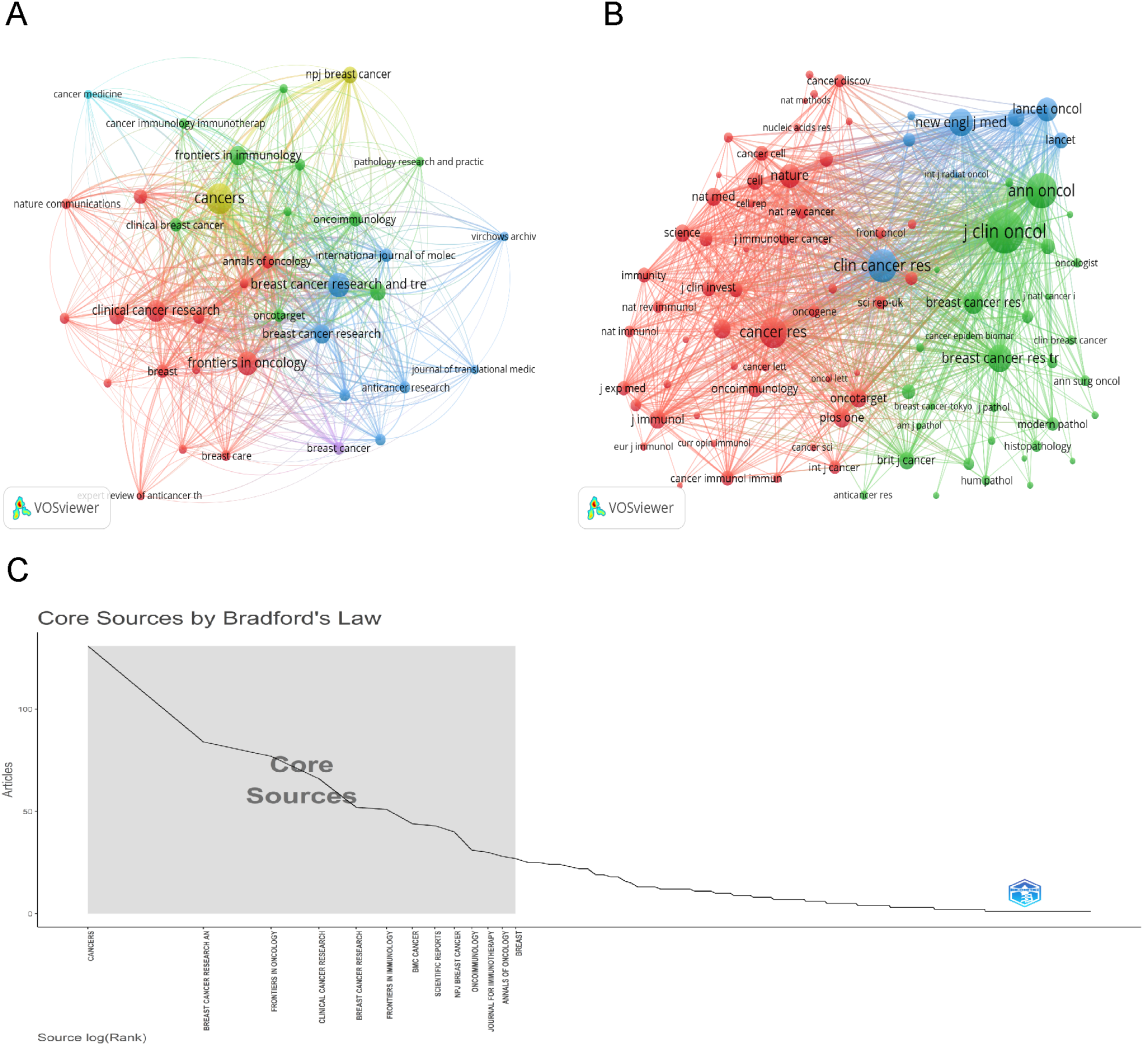
Visualization of Journals and Co-cited Journal Analysis. **(A)** Visualization of the cooperation network map of journals. **(B)** Visualization of the network map of co-cited journals. **(C)** Thirteen core sources by Bradford’s law.

After selecting 45 journals each with no fewer than 10 related articles, we mapped the journal network to display the interconnections between journal citations (**Figure 5A**). We also identified journals with at least 200 co-citations to delineate the co-citation network. As shown in **Figure 5B**, the interconnections of journal co-citations identified three clusters: cluster 1 (red) represented by CANCER RESEARCH and NATURE, cluster 2 (green) JOURNAL OF CLINICAL ONCOLOGY, ANNALS OF ONCOLOGY, and BREAST CANCER RESEARCH AND TREATMENT, and cluster 3 (blue) CLINICAL CANCER RESEARCH and NEW ENGLAND JOURNAL OF MEDICINE. Additionally, under Bradford’s Law, journals are divided into core, relevant, and non-relevant categories based on their focus on this subject, as depicted in **Figure 5C**. Of these, the 13 core journals published 704 articles, constituting 34.1% of the total.

Researchers monitor the development of concepts over time by analyzing citations and selecting the most relevant papers from comprehensive lists for their studies. Furthermore, the local citation score (LCS) measures the number of citations a document garners within a specific dataset. Evidently, articles with high citation counts offer significant insights into scientific progress (18). **Supplementary Table 2** details the top ten most-cited documents, predominantly published between 2014 and 2016. LOI S authored three papers, while SALGADO R authored two among them. The 2015 ANNALS OF ONCOLOGY publication by SALGADO R, “The evaluation of tumor-infiltrating lymphocytes (TILs) in breast cancer: recommendations by an International TILs Working Group 2014,” received the highest number of citations. This article reviews the clinical validity and utility of TILs in BC, aiming to enhance understanding in this rapidly evolving field. It proposes a standardized methodology for visual assessment of H&E-stained sections and recognizes the future potential of molecular and multiplexed approaches (7).

### Analysis of references/co-cited references

**Figure 6:**
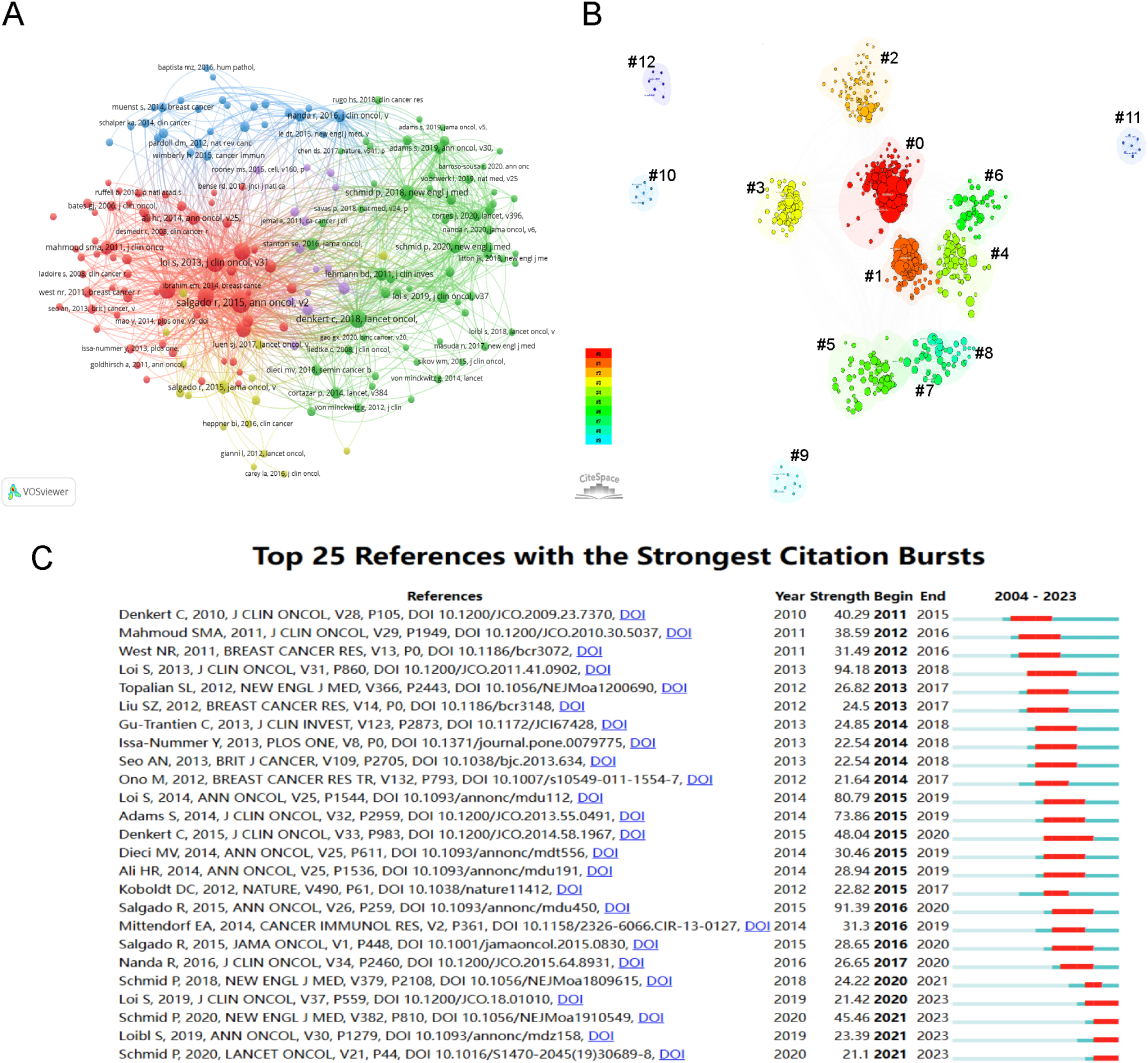
Visualization of Co-cited References and References Bursts. **(A)** Visualization of the network map of co-cited references. **(B)** Cluster visualization map of co-cited references (#0: tumor-infiltrating lymphocytes; #1: immunotherapy; #2: T cell; #3: PD-L1; #4: TNBC; #5: xCell; #6: HER2-positive breast cancer; #7: immune profile; #8: machine learning; #9: dendritic cell; #10: B7-H1; #11: tumor biology; #12: immunology/immunobiology). **(C)** Top 25 references with the strongest citation bursts.

From 2004 to 2023, scholarly references exploring tumor-infiltrating lymphocyte TILs in BC garnered 559,131 citations. Each of the top ten co-cited works, as listed in **Table 5**, received at least 250 co-citations. Subsequently, works with 50 or more co-citations were carefully selected to create the co-citation network diagram (**Figure 6A**). In this diagram, the size of each circle corresponds to the citation frequency, illustrating the scholarly impact of the works. For instance, significant co-citation activity was noted among prominent studies such as “SALGADO R, 2015, ANN ONCOL”, “LOI S, 2013, J CLIN ONCOL”, and “DENKERT C, 2018, LANCET ONCOL”. **Figure 6B** illustrates 13 distinct clusters identified using CiteSpace, covering topics such as “tumor-infiltrating lymphocytes”, “immunotherapy”, “T cell”, “programmed death ligand 1”, “triple-negative breast cancer”, “xCell”, “Her2-positive breast cancer”, “immune profile”, “machine learning”, “dendritic cell”, “B7-H1”, “tumor biology”, and “immunology/immunobiology”. The clustering is justified, evidenced by a Modularity Q value of 0.6426 and a Weighted Mean Silhouette score of 0.8747.

**Table 5:** Top 10 co-cited references with the highest local citations in the research field of TILs in BC.

Rank	Cited References (Author/Year/Journal/Volume/Page/DOI)	LC
1	SALGADO R, 2015, ANN ONCOL, V26, P259, DOI 10.1093/ANNONC/MDU450	833
2	LOI S, 2013, J CLIN ONCOL, V31, P860, DOI 10.1200/JCO.2011.41.0902	628
3	LOI S, 2014, ANN ONCOL, V25, P1544, DOI 10.1093/ANNONC/MDU112	516
4	DENKERT C, 2018, LANCET ONCOL, V19, P40, DOI 10.1016/S1470-2045(17)30904-X	515
5	DENKERT C, 2010, J CLIN ONCOL, V28, P105, DOI 10.1200/JCO.2009.23.7370	502
6	ADAMS S, 2014, J CLIN ONCOL, V32, P2959, DOI 10.1200/JCO.2013.55.0491	490
7	DENKERT C, 2015, J CLIN ONCOL, V33, P983, DOI 10.1200/JCO.2014.58.1967	408
8	SCHMID P, 2018, NEW ENGL J MED, V379, P2108, DOI 10.1056/NEJMOA1809615	377
9	MAHMOUD SMA, 2011, J CLIN ONCOL, V29, P1949, DOI 10.1200/JCO.2010.30.5037	257
10	NANDA R, 2016, J CLIN ONCOL, V34, P2460, DOI 10.1200/JCO.2015.64.8931	250

LC, local citations.

A burst citation refers to a reference frequently cited within a specific timeframe in a given field. Repeated citations of a set of articles lead to the formation of a conceptual cluster (19). In this study, CiteSpace identified 25 references with strong burst citations. **Figure 6C** shows the references arranged by burst sequence and initial publication years, with each bar representing a year. The red lines indicate a sudden increase in highly-cited references within a specific year. Authored by Loi S, the study “Prognostic and Predictive Value of Tumor-Infiltrating Lymphocytes in a Phase III Randomized Adjuvant Breast Cancer Trial in Node-Positive Breast Cancer Comparing the Addition of Docetaxel to Doxorubicin With Doxorubicin-Based Chemotherapy: BIG 02-98,” (20) recorded the highest citation burst (intensity=94.18) from 2013 to 2018. The article, “Tumor infiltrating lymphocytes are prognostic in triple negative breast cancer and predictive for trastuzumab benefit in early breast cancer: results from the FinHER trial,” (21) third in burst strength (80.79) and authored by Loi S, experienced a citation burst from 2015 to 2019. These two references primarily discuss the clinical implications of TILs in BC, particularly their prognostic and predictive roles in triple-negative and HER2-positive subtypes, as well as standardized methodology for its evaluation. The 2014 study, “The evaluation of tumor-infiltrating lymphocytes (TILs) in breast cancer: recommendations by an International TILs Working Group 2014,” (7) shows the second-highest citation burst (intensity=91.39) from 2016 to 2020.

### Analysis of keywords and hotspots

**Figure 7:**
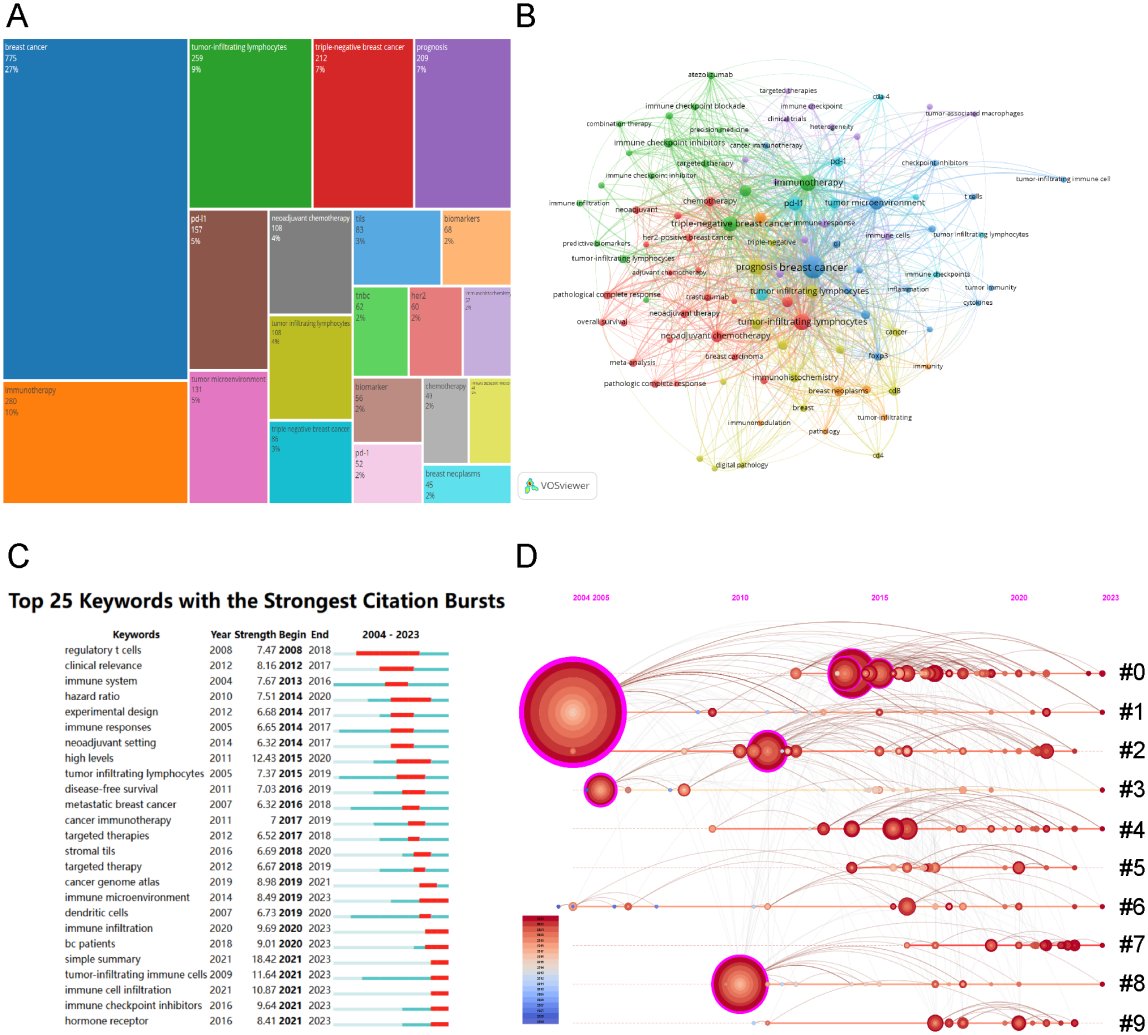
Visualization of Keywords Analysis. **(A)** Keyword tree-map of the retrieved articles (top 20 frequent keywords). **(B)**Visualization of the cooperation network map of keywords’ co-occurrence. **(C)** Top 25 keywords with the strongest citation bursts. **(D)** Timeline visualization of keywords cluster analysis (#0: neoadjuvant chemotherapy; #1: breast cancer; #2: tumor microenvironment; #3: tumor infiltrating; #4: breast neoplasms; #5: immune cells; #6: tumor-infiltrating lymphocytes; #7: machine learning; #8: tumor-infiltrating immune cells; #9: immune checkpoint inhibitors).

Keywords act as succinct indicators of specific topics, providing a comprehensive overview of related literature. High-frequency keywords identify research hotspots and key issues within a discipline. This study analyzed 2,858 keywords proposed by the authors. The tool Bibliometrix visualized keyword occurrences and frequencies. **Figure 7A** presents a tree-map of keywords from the retrieved articles, where area of the rectangle corresponds to keyword frequency. Leading keywords were “breast cancer”, “immunotherapy”, and “tumor-infiltrating lymphocytes”, succeeded by “triple-negative breast cancer”, “prognosis”, “PD-L1”, “tumor microenvironment”, “neoadjuvant chemotherapy”, and “biomarkers”. **Figure 7B** shows the most frequently used keywords, as identified by co-occurrence analysis using VOSviewer. Node proximity reflects the frequency of keyword co-occurrence. The keywords “breast cancer”, “immunotherapy”, and “tumor-infiltrating lymphocytes” showed the highest co-occurrence rates and the strongest links to other terms.

Employing a keyword co-occurrence network, we performed a burst analysis to identify the 25 keywords with the most significant citation bursts, as shown in **Figure 7C**. The analysis indicated that “simple summary” (18.42) exhibited the strongest burst, followed by “high levels” (12.43) and “tumor-infiltrating immune cells” (11.64). Additionally, keywords including “immune cell infiltration” (10.87), “immune checkpoint inhibitors” (9.64), and “hormone receptor” (8.41) were identified as recent bursts from 2021 to 2023, signaling emerging research hotspots. The cluster analysis of keywords on the timeline (**Figure 7D**) shows that immune checkpoint inhibitors for BC immunotherapy and deep learning applications on TILs in BC are current research trends.

**Figure 8:**
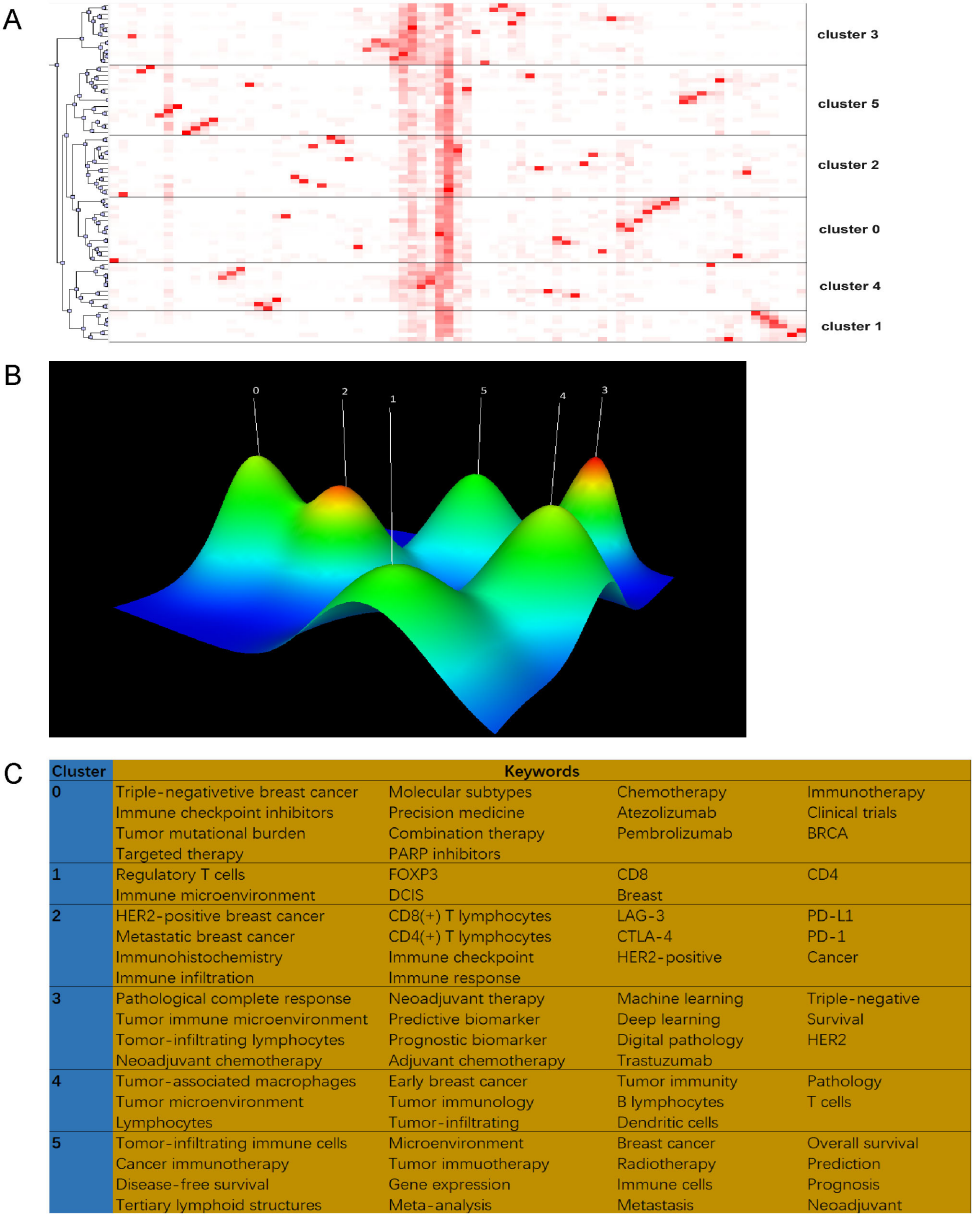
Biclustering analysis of 77 high-frequency keywords. **(A)** Matrix visualization of binary matrix bicluster analysis of keywords. **(B)** Mountain visualization of binary matrix bicluster analysis of keywords. **(C)** High frequency keywords in each cluster.

We employed biclustering analysis with BICOMB and gCLUTO to delineate the identified research hotspots. BICOMB produced a co-keyword matrix (**Figure 8A**), which was further analyzed by gCLUTO, resulting in a mountain graph (**Figure 8B**) that disclosed six distinct clusters in the research field. **Figure 8A** categorizes the 77 high-frequency keywords (each appearing at least 10 times) into six groups. In **Figure 8B**, the gaps between mountains reflect the correlation levels among clusters, whereas the height and volume of each mountain indicate the internal similarity and term coverage, respectively. Additionally, the peak’s color gradient from red to green represents the standard deviation, emphasizing the variation levels. The analysis pinpointed six key hotspots: (I) TILs in TNBC immunotherapy; (II) TILs in DCIS; (III) TILs in HER2-positive BC immunotherapy; (IV) AI in pathological assessment of TILs; (V) TILs in early BC microenvironment; (VI) Prognostic and predictive roles of TILs in BC. **Figure 8C** visualizes the distribution of 64 high-frequency keywords across each cluster.

## Discussion

Research on tumor-infiltrating lymphocytes (TILs) in breast cancer (BC) has shown a link between pathologically assessed TILs and clinical outcomes, with differences noted among various subtypes (22–24). Although there is a wealth of literature available, scientometric analyses and visualizations remain scarce. This research presents the inaugural comprehensive bibliometric assessment to examine research trends and emerging topics related to TILs in BC. We examined 22,066 publications from 22004 to 2023, derived from the Web of Science Core Collection, employing bibliometric tools (Bibliometricx in R, CiteSpace, VOSviewer) alongside biclustering methods (BICOMB, gCLUTO). This method enabled the visualization of multiple dimensions such as yearly trends, institutional affiliations, authorship patterns, publication venues, co-citation networks, references, and keyword usage.

### General information

Between 2004 and 2013, fewer than 30 articles were published annually in this field. Since 2014, publication numbers have increased continuously, reflecting growing interest and recognition within the scientific community. Contributions to this field came from 112,069 co-authors across 886 countries, with the USA, China, and Italy as the leading contributors. The USA dominates this research area globally, leading in total publications, citation counts, and network link strength. Despite China’s significant increase in publication output, ranking second globally, it lags in producing cooperative high-impact research. Institutional analysis shows that Milan University leads the top 10 institutions in article count. According to Bradford’s Law, **Figure 5C** lists 13 core journals identified as significant contributors in journal analysis. Among them, CANCERS published the most articles, JOURNAL OF CLINICAL ONCOLOGY was the most cited, and CLINICAL CANCER RESEARCH had the highest impact factor. This analysis offers crucial guidance for researchers in choosing suitable publication venues.

This research highlighted the leading authors in the field, ranking them by publication count and H-index. LOI S of the Peter Maccallum Cancer Center emerged as the top-ranked author in these categories, closely followed by Salgado R of Universite libre de Bruxelles. LOI S authored three of the top 10 most cited articles globally and locally. In her publication from the BIG 02-98 study, LOI S demonstrated that increased lymphocytic infiltration correlates with better prognosis and enhanced efficacy of anthracycline-only chemotherapy in node-positive, ER-negative/HER2-negative BC (20). Additionally, her FinHER trial revealed that higher initial levels of TILs significantly correlate with lower distant recurrence rates in primary TNBC (21). The FinHER trial also first reported that elevated TIL levels enhance the efficacy of trastuzumab in HER2-positive conditions (21). In 2019, LOI S conducted a pooled analysis confirming TILs’ strong prognostic value in early-stage TNBC, showing that high TILs levels post-adjuvant chemotherapy correlate with excellent patient survival (25). This supported incorporating TILs into the clinicopathologic prognostic models for TNBC. These studies set a new standard for understanding the impact of TILs on various BC subtypes. The highest cited paper, co-authored by LOI S and Salgado R, introduced a standardized approach for the pathological evaluation of TILs, facilitating their integration into routine histopathological analysis (7).

### Hotspots and trends

Joint analysis of references and keywords reveals trends in current and future research. The 2015 study by Salgado R, “The evaluation of tumor-infiltrating lymphocytes (TILs) in breast cancer: recommendations by an International TILs Working Group 2014,” recognized for its citation burst in recent years, assessed the clinical relevance and utility of TILs in BC. This work aimed to enhance understanding in this dynamic area, establish a standardized visual assessment protocol for H&E stained sections, and recognized the emerging potential of molecular and multiplexed techniques (7). Trending term analysis reveals a knowledge shift regarding TILs in BC, moving from classic treatments such as chemotherapy and targeted therapy to combination with immunotherapy. The consensus among researchers and clinicians now emphasizes the significance of TILs across various stages and subtypes of BC, their impact on the immune microenvironment, and their efficacy in combined immunotherapy. Keyword co-occurrence and bicluster analysis have identified six primary research topics, with *cluster 3* and *5* being particularly prominent.

#### Cluster 0: TILs in TNBC immunotherapy

TNBC’s microenvironment is densely populated by TILs, which inherently exhibit immunogenic properties (26, 27). TNBC is the most extensively studied BC subtype due to its high immunogenicity and elevated levels of TILs linked to favorable prognosis (28). Additionally, TNBCs show significant CD8+ and CD4+ T-cell infiltration in stromal and intratumoral regions, compared to hormonal receptor (HR)-positive tumors.

Managing TNBC increasingly relies on ICIs (29). In early-stage TNBC, ICIs show promising outcomes when used in combination with chemotherapy. Recent investigations such as KEYNOTE-522, IMpassion031, and GeparNUEVO (30), demonstrate that neoadjuvant treatment involving both immunotherapy and chemotherapy provides synergistic advantages. The levels of TILs in metastatic triple-negative breast cancer (mTNBC) serve as indicators of the likelihood of patients responding to immunotherapy. This was validated by the KEYNOTE-086 study, which included 228 mTNBC patients with different PD-L1 expressions treated with pembrolizumab monotherapy. Higher sTIL levels (≥ 10%) correlated with better objective response rates (ORRs) in comparison to lower levels (< 10%) (31). Another study examines the effect of autologous TIL therapy in pretreated metastatic TNBC patients. Immunotherapy, particularly ICIs, is likely to become the standard treatment for TNBC in the future, as evidenced by the current findings. Considering the demonstrated benefits of TILs in advanced cancers, this research seeks to develop superior treatment options for metastatic TNBC (32). Consequently, pinpointing appropriate biomarkers to forecast patient response to immunotherapy is crucial. TILs concentration correlates with elevated PD-L1 expression and positive immunotherapy outcomes (33, 34). TILs count serves as a predictive indicator for immune checkpoint blockade (ICB) efficacy (35). High TILs infiltration, coupled with increased PD-L1 expression, foretells a better response to pembrolizumab in patients with advanced and metastatic TNBC (34, 36). Additionally, in patients with high PD-L1 expression and TILs infiltration (≥5%), pembrolizumab led to superior survival outcomes as compared to chemotherapy. Furthermore, higher TIL infiltration (≥5%) was linked with enhanced PCR and OS benefits in patients receiving atezolizumab (34). The data suggests that increased TILs infiltration amplifies anti-tumor immune responses, resulting in more effective cancer cell eradication. These findings propose that TILs might predict response to ICI therapy, though their interpretation should be approached cautiously.

Mechanisms contributing to the important role in TILs in immunotherapy in BC may involve the following aspects. Immune surveillance and tumor recognition: Immune surveillance is a fundamental concept in tumor immunology, referring to the immune system’s ability to recognize and eliminate tumor cells. In TNBC, TILs are believed to play a crucial role in this process. These lymphocytes can identify tumor-specific antigens and eradicate cancer cells through the release of cytokines and cytotoxic mechanisms. The composition of TIL subpopulations, including CD8+ T cells and regulatory T cells (Tregs), significantly influences the effectiveness of immune surveillance. For patients with TNBC, enhanced immune surveillance may aid in controlling tumor progression and provide a foundation for subsequent immunotherapy (37). Cytokine production: The production of cytokines is one of the critical mechanisms by which TILs contribute to the immune response in TNBC. TILs enhance anti-tumor immune responses by producing various cytokines, such as interferon-γ and tumor necrosis factor-α. These cytokines not only directly inhibit the growth of tumor cells but also promote the recruitment and activation of other immune cells. For instance, interferon-γ enhances the antigen-presenting capacity of tumor cells, thereby improving T cell recognition (38). In TNBC, the level of cytokine production by TILs correlates closely with the patient’s treatment response and prognosis. Consequently, assessing the cytokine-generating capability of TILs may provide novel biomarkers for personalized immunotherapy (39). Immune checkpoint modulation: Immune checkpoints play a pivotal role in regulating the function of TILs. In TNBC, the activation of the PD-1/PD-L1 pathway often leads to functional exhaustion of TILs, thereby inhibiting their anti-tumor activity. Research has shown that blocking the PD-1/PD-L1 pathway can restore the cytotoxic functions of TILs and enhance the immune response against tumors (38). Additionally, the modulation of other immune checkpoints, such as CTLA-4, is also believed to impact TIL function. Thus, the combined use of ICIs may represent an effective therapeutic strategy to enhance the immune response in TNBC patients (40). Tumor microenvironment (TME) interaction: TME significantly influences the function and activity of TILs. In TNBC, cellular components within the TME, such as tumor-associated macrophages and fibroblasts, as well as their secreted cytokines and chemokines, can modulate the infiltration and activation status of TILs. Studies indicate that the immunosuppressive characteristics of the TME may lead to a decline in TIL function, thereby affecting the efficacy of immunotherapy (41). Furthermore, the interactions between TILs and the TME may also promote tumor progression. Therefore, interventions targeting the TME could provide new strategies to enhance the anti-tumor effects of TILs (42). Clonal expansion and memory formation are essential mechanisms through which TILs exert long-lasting anti-tumor effects in TNBC. During the immune response, specific T cell clones can enhance long-term surveillance against tumors by proliferating and forming memory cells. Research indicates that successful clonal expansion of TILs correlates closely with the intensity of the anti-tumor response (43). The formation of memory T cells enables the body to mount a rapid and robust immune response upon re-encountering tumor antigens, which is crucial for preventing tumor relapse. Therefore, promoting the clonal expansion and memory formation of TILs may offer novel strategies for immunotherapy in TNBC patients (44).

#### Cluster 1: TILs in DCIS

Although ductal carcinoma *in situ* (DCIS) is a non-invasive tumor, it can display aggressive clinical behavior such as a high incidence of local recurrence and progression to invasive cancer. Consequently, it is crucial to identify prognostic factors for DCIS. While the highest density of immune cells is found in invasive breast cancer, the most notable change is observed between normal adjacent tissue and DCIS (45). This suggests that immune cells are present as early as the *in situ* stage of cancer development. Recent studies have concentrated on TILs within the tumor microenvironment. The main components of TILs in DCIS, listed in decreasing order, are T cells (CD3+, CD8+, FOXP3+), followed by B cells (CD20+) (46). The methods for assessing TILs in DCIS are still undefined, creating challenges for reproducibility and interpretation of prognostic significance. An investigation of 534 DCIS cases discovered that a high density of TILs was linked to larger tumor size, comedo-type necrosis, intermediate to high grades, concurrent Paget’s disease, lack of ER expression, younger age, and shorter recurrence-free periods (47). In a group of 283 DCIS cases, patients having TILs over 17% demonstrated a higher risk of recurrence. Additionally, a meta-analysis of seven studies including 3437 DCIS cases showed that elevated TILs were associated with triple-negative and HER2+ phenotypes, higher grade, necrosis, and an increased risk of both invasive and non-invasive recurrence (48). A thoroughly characterized cohort (n=700) of pure DCIS (n=508) and DCIS with invasive carcinoma, followed for the long term, indicated that high-density tumor-infiltrating lymphocytes (TILs), stromal FOXP3, and PDL1 are poor prognostic factors for DCIS recurrence. Moreover, high-density TILs independently correlate with poor outcomes for all forms of recurrence, particularly invasive recurrence (49). Knoepfelmacher et al. found an association between elevated DCIS-associated TILs and higher oncotype DX scores for DCIS recurrence. A high oncotype DX score was strongly correlated with a higher tumor grade (50). Conversely, an analysis of 1488 DCIS patients found that higher TILs were linked to HER2+ phenotype, higher grade, and necrosis, but had no effect on ipsilateral DCIS or invasive tumor recurrence, regardless of treatment approach (51). Recently, Miligy et al. acknowledged a significant connection between low B cell count and prolonged recurrence-free survival. This link was particularly noted in B cells adjacent to DCIS (52).

#### Cluster 2: TILs in HER2-positive BC immunotherapy

HER2 amplification induces a non-inflamed TME with fewer TILs compared to TNBC (53). Evidence supports the inherent immunogenicity of HER2-positive tumors, highlighting the roles of adaptive immune responses via T and B lymphocytes. In the HER2-positive tumor immune microenvironment (TIME), the HER2 protein is a specific antigen recognized by T and B cells, suitable for immunotherapy (54).

Immunogenicity data for HER2-positive BC have led to clinical trials of ICIs, generally yielding poor results. The PANACEA trial studied trastuzumab and pembrolizumab in patients with HER2-positive metastatic BC progressing on trastuzumab; PD-L1-positive patients showed a 15.2% objective response rate (ORR), while PD-L1-negative patients had 0%. TILs were common in PD-L1-positive patients and correlated with ORR, suggesting these patients might benefit from trastuzumab-ICI therapy (55). Meanwhile, the KATE2 trial showed that combining atezolizumab with T-DM1 did not improve progression-free survival (PFS) or overall survival (OS) universally. Interestingly, high TILs patients (≥5%) had longer PFS with atezolizumab, contrasting with shorter PFS in the placebo group (56). Controversial data from metastatic settings require further scrutiny due to variances in TILs thresholds, sampling sites, and patient profiles.

#### Cluster 3: AI in pathological assessment of TILs

In 2014, detailed guidelines were issued by the International TILs Working Group to standardize TILs assessment in breast cancer (7). The aim of these guidelines was to ensure reproducible and accurate evaluations, emphasizing the need for a standardized methodology. To preserve uniformity in different research and clinical settings, these guidelines specified parameters, scoring systems, and reporting criteria for TILs analysis. Artificial intelligence (AI) technologies, including machine learning, deep learning, neural networks, natural language processing, cognitive computing, and computer vision, provide innovative tools for pathologists to handle new assessments. Furthermore, these algorithms may replace some expensive molecular tests in breast pathology (57). Recognized as vital prognostic biomarkers for TNBC, TILs need to be accurately quantified to enhance the understanding and management of TNBC (58, 59). Balkenhol et al. examined different objective methodologies for assessing TILs in immunohistochemically stained sections, correlating these assessments with patient outcomes (60). They employed automated deep learning to analyze CD3, CD8, and FOXP3 markers across various tumor regions. The findings indicated a consistent negative correlation between the abundance of TILs and both recurrence-free survival (RFS) and OS, irrespective of analyzed markers.

Recently, Sangjoon C et al. developed a deep learning (DL) analyzer for assessing stromal tumor-infiltrating lymphocytes (sTILs) in breast cancer using 402 whole slide images, interpreted by three pathologists (61). The DL model’s performance was evaluated in 210 cases with sTIL scores differing by less than 10 percentage points from the pathologists’ scores. In patients with triple-negative and HER2-positive BC receiving neoadjuvant chemotherapy, DL-assisted analysis showed higher sTIL scores in responders and a correlation between high sTIL levels (sTIL ≥ 50) and chemotherapeutic response. Concurrently, a supervised DL model analyzed H&E-stained WSIs for TILs in 2231 early-stage luminal BC patients with extended follow-up (62). Results indicated AI-based evaluation of the stromal proportion of TILs did not predict patient outcomes.

#### Cluster 4: TILs in early BC microenvironment

Research indicates a progressive rise in immune cell counts from normal breast tissue to invasive breast cancer (45, 63). TILs prevalence is influenced by tumor stage and metastatic sites, with early-stage breast cancer showing the highest infiltration rates (64). Tumor immunity emerges from complex interactions among immune cells, their mediators, cancer cells, and the microenvironment, maintaining a dynamic balance. Various immune cell subtypes, including CD8+ cytotoxic T lymphocytes, Th1 lymphocytes, and M1 phenotype tumor-associated macrophages (TAMs), play distinct roles in the antitumor response (65–68). CD8+ T cell infiltration, critical in some BC cases, has been observed through immunohistochemistry (66). T-follicular helper (Tfh) cells and Natural killer (NK) T cells also contribute significantly to antitumor immunity (69–71). Tfh cells, located in germinal centers of tertiary lymphoid structures, aid in the selection and maintenance of B cells, leading to antibody production. The resulting type 1 cytokine-predominant response primarily involves IFN-γ, TNF, and IL-2. Conversely, FOXP3+ regulatory T (Treg) cells, acting as immune suppressors, impact high-avidity CD8+ T cell selection and functional inhibition of various immune cells, including antigen-presenting cells (APCs), Th1, CD8+ T, and NK cells.

#### Cluster 5: Prognostic and predictive roles of TILs in BC

The roles of TILs in prognosis and prediction vary among BC subtypes. In TNBC, the prognostic significance of stromal TILs (sTILs) has been evaluated across stages. An analysis of 2148 early-stage TNBC patients, with an average sTIL level of 23%, demonstrated that 55.8% received anthracycline regimens and 44.2% combined with taxane. Elevated sTILs correlated with better prognoses, with HRs of 0.87 [invasive disease-free survival (iDFS)], 0.83 [distant disease-free survival (dDFS)], and 0.84 (OS) per 10% increase in sTILs (25). Patients with sTILs ≥ 30% and no lymph node metastases had significantly better outcomes. These findings contributed to prognostic model development incorporating sTILs. A SABCS (2023) study on 134 stage I-III TNBC patients who achieved pCR after NAChT showed better 5-year relapse-free survival (RFS) and OS for those with baseline TILs >20%. Research indicates a positive correlation between baseline sTIL levels and tumor response in neoadjuvant chemotherapy cohorts. The GeparNuevo study, involving 174 TNBC patients, found better iDFS in those with TILs ≥11% (72). CD8+ lymphocytes in TILs subsets are linked to improved survival in the basal-like subgroup and favorable therapeutic responses (66, 73, 74), while CD4+ and FOXP3+ infiltrates’ prognostic roles are ambiguous (63, 75). Most studies identify TILs as positive prognostic markers in TNBC, likely due to the dominance of CD8+ T cells (73). TILs are crucial for selecting patients for de-escalated systemic therapies, given their association with better chemo-immunotherapy responses, pCR rates, and overall prognosis.

HER2-positive breast cancer’s heterogeneity results in diverse TILs levels among molecular subtypes, with HER2-enriched tumors having the highest immune infiltration (76). Studies reveal a positive correlation between higher TILs levels and better responses to neoadjuvant therapy and clinical outcomes in early HER2-positive BC. Elevated TILs levels correlate with increased pCR rates and enhanced DFS (77, 78). The FinHER trial shows similar positive associations for adjuvant settings involving trastuzumab (21). The Phase III APHINITY trial indicates that high TILs levels and T-cell-related genes predict improved invasive DFS with dual blockade (pertuzumab and trastuzumab). Notably, TILs are dynamic biomarkers, effective during treatment as shown in the PAMELA trial, only TILs measured during treatment were associated with a pCR, not those measured at baseline (76, 79). However, their prognostic and predictive roles in advanced diseases are vague. A CLEOPATRA trial retrospective analysis connects higher pre-treatment sTILs levels with increased OS, regardless of treatment type.

HR+/HER2- BC is the least immunogenic subtype, typically exhibiting fewer TILs. Its TME is highly heterogeneous, and the prognostic value of TILs is debated based on extensive studies. In a cohort of 2231 early-stage luminal BC patients, higher sTIL and tTIL counts were linked to poorer clinical outcomes such as higher tumor grade, lymph node metastasis, larger tumors, and younger age (62). A meta-analysis of 2836 patients found that higher TILs levels correlated with reduced OS in HR+/HER2- early BC patients receiving neoadjuvant chemotherapy. On the other hand, the German Breast Cancer Group’s pooled analysis further revealed a strong association between TIL levels and pCR rates: 6% for low TILs, 11% for intermediate TILs, and 28% for high TILs (80).

Radiation therapy (RT) is pivotal in curing BC, yet the role of TILs in RT response remains unclear for invasive BC patients. The SweBCG91 RT trial, involving 1178 stage I-II BC patients, examined IBTR over 10 years and found 71% with low TILs tumors benefited from RT (81). A Danish study using DBCG82bc trial data linked high TILs with reduced distant metastasis (DM) risk and better OS, without a connection to loco-regional control (82). A Detroit retrospective study (2009–2019) reported that patients with low TILs saw no RT benefit post-lumpectomy, but high TILs patients had improved DFS and OS with lumpectomy and RT (83). Study differences might owe to factors like varying TILs cut-off points, differing patient profiles and treatments, and study power for TILs and RT-response interactions.

### Strengths and limitations

This is the first systematic bibliometric analysis of TILs in BC. It provides essential insights for collaboration and offers guidance to both researchers and clinicians. In summary, the analysis has revealed significant trends in the study of TILs in BC, particularly emphasizing their role in various subtypes, notably TNBC and HER2-positive BC. Despite the advancements, several weaknesses persist in the current understanding of TILs in BC: Standardization Issues: While guidelines for TIL assessment have been established, discrepancies in methodologies and scoring systems remain, complicating the interpretation of results across studies. Lack of Clarity in Non-TNBC Subtypes: The role of TILs in hormone receptor-positive (HR+) and HER2-negative BC is less clear, with conflicting evidence regarding their prognostic significance. The heterogeneity of the immune microenvironment in these subtypes poses challenges for consistent findings. Variability in TIL Assessment: Variations in TIL assessment methods, including differences in cut-off values and sampling techniques, lead to inconsistencies in results and hinder the establishment of universally applicable biomarkers. The field of TILs in BC is poised for several promising research directions: Biomarker Development: Future studies should focus on identifying robust biomarkers that can predict patient responses to immunotherapy, particularly in TNBC and HER2-positive subtypes. The relationship between TILs, PD-L1 expression, and treatment outcomes should be further elucidated. Mechanistic Insights: Investigating the underlying mechanisms by which TILs influence tumor progression and response to therapy will enhance the understanding of their role in the tumor microenvironment. This includes exploring cytokine production, immune checkpoint modulation, and interactions with other immune cells. Personalized Immunotherapy: Research should aim to develop personalized immunotherapy strategies based on TIL profiles, integrating genomic and transcriptomic data to tailor treatments for individual patients. Integration of AI Technologies: Continued integration of AI and machine learning in the analysis of TILs will improve the accuracy of assessments and potentially reduce reliance on traditional, more expensive molecular testing. Prospective Clinical Trials: There is a critical need for well-designed prospective clinical trials that assess the impact of TIL levels on treatment outcomes across various BC subtypes, particularly focusing on the dynamic nature of TILs during therapy.

However, our study has some limitations methodologically. Specifically, it solely utilized data from WoSCC, potentially overlooking articles from other databases. Furthermore, the bibliometric tools used, like VOSviewer and CiteSpace, could introduce bias and variability due to different software versions. We used citation frequency as a quality indicator, though it can be influenced by the article’s age. The scope of the analysis was limited to publications from January 2004 to December 2023. Consequently, newer studies published after December 2023 were not included, potentially introducing additional bias.

## Conclusion

This study presents an extensive and insightful analysis of Tumor-Infiltrating Lymphocytes (TILs) in breast cancer (BC) using visual and bibliometric methods. The body of literature in this field is rapidly expanding, with substantial contributions from researchers in the United States. We identified and reviewed six major research hotspots within this domain. Specifically, the prognostic and predictive roles of TILs in triple-negative breast cancer (TNBC) and HER2-positive BC show strong impacts in clinical practice. However, accurately determining TILs status using artificial intelligence (AI) and evaluating its prognostic and predictive value in estrogen receptor-positive (ER-positive) BC remain significant challenges. As immunotherapy continues to progress, the significance of TILs in treatment planning will increasingly become prominent. This study could be an essential resource for researchers worldwide.

## Data Availability

The original contributions presented in the study are included in the article/**Supplementary Material**. Further inquiries can be directed to the corresponding author.
